# Supplementary material for: Mediterranean diet effects on vascular health and serum levels of adipokines and ceramides
Source: PLoS One. 2024 May 29;19(5):e0300844. doi: 10.1371/journal.pone.0300844 (PMC11135776; doi:10.1371/journal.pone.0300844)
Supplement: S1 File — (PDF) [file pone.0300844.s001.pdf]

Mediterranean diet effects on vascular health and serum levels of adipokines and ceramides. A Randomized Trial. *Trial protocol*

Background

Current knowledge of complex interplays between nutrients and other food constituents has driven to a better knowledge of the concept of total dietary patterns. The concept of a "dietary scheme" has been progressively used to involve a complete and exhaustive analysis of food and nutrient consumption in particular dietary pattern.

Current knowledge highlights the crucial role of the Mediterranean-style diet in the prevention of cardiovascular diseases. The Lyon diet heart study reported the beneficial effects of the Mediterranean-style diet on the secondary prevention of cardiovascular diseases (4).

The PREDIMED (Prevention with Mediterranean Diet) study is the first large-scale randomised trial that reported the effectiveness of the Mediterranean-style diet in primary prevention in terms of reduction of incidence cardiovascular events, such as strokes, atrial fibrillation, peripheral vascular disease, and myocardial infarction .

This study showed that adherence to the Mediterranean-style diet supplemented with extra virgin olive oil or walnuts obtained a significant reduction of cardiovascular events in comparison to the control group randomized to a low-fat diet.

Cardiovascular disease, which includes coronary artery disease, cerebrovascular disease, peripheral artery disease and heart failure, is the leading cause of mortality worldwide. Atherosclerosis is the main cause of cardiovascular diseases, particularly coronary artery disease and stroke; it is a diasese with a possible genetic , metabolic, environmental and behavioural based-pathogenesis.

Hypercholesterolemia is the main metabolic risk factor of atherosclerosis . Seveeral studies have reported that reducing fat in diet reduces the levels plasma cholesterol and it is associated to a lower incidence of coronary heart disease; this effect has been reported in particular with saturated fat reduction and with its replacing with polyinsaturated and monounsaturated fat of virgin olive oil.

However, the need for additional predictive biomarkers is underlined by the very substantial residual risk that exists above the standard clinical and biochemical risk predictors. Ceramides are members of the sphingolipid family and precursors of complex sphingolipids. Some studies in cultured cells and animal models repprted that the accumulation of ceramides may lead to the activation of pathologifc pathways altering normal cellular function, including the action of insulin Thus the excess de novo ceramide biosynthesis may be due to cellular stress stimuli, suche as high serum levels of saturated free fatty acids. Ceramide and its metabolites represent an intermediate link between over-nutrition and the pathogenetic basis of the cardiometabolic disease risk, including insulin resistance and low-grade inflammation. Thus it appears worthy of interest the evaluation of the role of serum ceramide leves as a expression of the interplay between lipidaemic pathways and vascular health status.

Adipocytes produce some hormones that directly interplay with the local microenvironment as well as distant tissues. Thus adipocytes have a possible pathogenetic role in the development of cardiometabolic diseases. Some adipokines produced by adipose tissue have been reported to be linked to inflammation and atherosclerosis and its related cardiovascular complications .

Few studies have prospectively analyzed the association between ceramides and the incidence of cardiovascular and cerebrovascular events. Laaksonen et al. reported a not well clear relationship of plasma ceramides with death from cardiovascular disease (CVD). It has been also suggested a possible role of the ratio of two ceramides as a possible predictive factor cardiovascular risk . The PREDIMED study suggested that dietary interventions according a Mediterranean-style diet positively modified the association between a blood ceramide score (comprised of individual ceramides and ceramide ratios) and risk of incident CVD.

Furthermore, The CORDIOPREV in patients with established coronary heart disease randomly to receive a Mediterranean diet or a low-fat diet intervention, with a follow-up of 7 years analyzed the primary outcome of a composite of major cardiovascular events, including myocardial infarction, revascularisation, ischaemic stroke, peripheral artery disease, and cardiovascular death. Authors

concluded that in secondary prevention, the Mediterranean diet was superior to the low-fat diet in preventing major cardiovascular events.

Nevertheless no study have evaluated in the same study the effects of Mediterranean Diet on endothelial function in parallel to its effects on serum lipid change including serum ceramide changes and on some adipokine serum pathway.

On this basis we designed a randomized trial to evaluate the effects of Mediterranean diet on endothelial function measured by the evaluation of reactive hyperaemia index (RHI) and the diet effects on serum ceramide and adipokines levels.

Methods

Patients and recruitment

consecutive patients at high risk of cardiovascular diseases admitted to the Internal Medicine and Stroke Care ward at the University Hospital of Palermo between September 2017 and December 2020 will be enrolled

The enrolled patients will be male subjects aged between 55 and 80 and female subjects between the ages of 60 and 80 who tested positive for at least two of the following eligibility criteria thus to be classified as subjects at high cardiovascular risk

1) Type 2 diabetes mellitus
2) Arterial hypertension
3) Body mass index (BMI) ≥ 25
4) Active smoking
5) Family history of early cardiovascular disease
6) Previous cardiovascular or cerebrovascular events (> 6 months)

Exclusion criteria

All patients with recent (<6 months) cardiovascular or cerebrovascular events were excluded

Randomization

The enrolled subjects, after the evaluation of the degree of adherence to a dietary regimen of the Mediterranean-style diet, were randomised

to two different types of dietary schemes:

1. Group A (experimental arm) - Mediterranean Diet : adherence to a Mediterranean-style diet was assessed through dietary screening at each follow up visit (every three months) for the entire duration of the study (twelve months).

2. Group B (control arm) - Low-fat diet: for which the enrolled subjects received dietary "counselling" starting from their first visit at the time of enrolment and, subsequently, every three months for the entire duration of the study (twelve months).

The randomization code was based on computer-generated random numbers and a 2:1 randomization ratio for experimental/control arm was performed both to contain the high cost of clinical investigations to be performed, and to avoid the effects of a high drop out rate by allowing more power for a per-protocol analysis. As a consequence of the type of randomization chosen, in order to maintain the power of the study, an increase in sample size of 12% was planned.
The investigators involved in clinical data collection and the measurement of outcome variables were not directly involved in the patients' treatment and were masked to the randomization process. The randomization code was maintained only at the central data facility and was not broken until all data analysis was complete

Dietary Intervention Trial

We will adopt the dietary intervention protocol with regard of Mediterranean Diet and low-fat diet validated by the PREDIMED trial (5).

A. Mediterranean Diet : The general guidelines to follow the Mediterranean diet that researchers will provide to participants included the following positive recommendations: a) abundant use of olive oil for cooking and dressing dishes; b) consumption of ≥ 2 daily servings of vegetables (at least one of them as fresh vegetables in a salad), discounting side dishes; c) ≥ 2-3 daily servings of fresh fruits (including natural juices); d) ≥ 3 weekly servings of legumes; e) ≥ 3 weekly servings of fish or seafood (at least one serving of fatty fish); f) ≥ 1 weekly serving of nuts or seeds; g) select white meats (poultry without skin or rabbit) instead of red meats or processed meats (burgers, sausages); h) cook regularly (at least twice a week) with tomato, garlic and onion adding or not other aromatic herbs, and dress vegetables, pasta, rice and other dishes with tomato, garlic and onion adding or not aromatic herbs. This sauce is made by slowly simmering the minced ingredients with abundant olive oil. Negative recommendations are also given to eliminate or limit the consumption of cream, butter, margarine, cold meat, pate, duck, carbonated and/or sugared beverages, pastries, industrial bakery products (such as cakes, donuts, or cookies), industrial desserts (puddings, custard), French fries or potato chips, and out-of-home pre-cooked cakes and sweets. The researchers insisted that two main meals per day should be eaten (seated at a table, lasting more than 20 minutes). For usual drinkers, the advice was to use wine as the main source of alcohol (maximum 300 ml, 1-3 glasses of wine per day). If wine intake was customary, a recommendation to drink a glass of wine per day (bigger for men, 150 ml, than for women, 100 ml) during meals was given. Ad libitum consumption was allowed for the following food items: nuts (raw and unsalted), eggs, fish (recommended for daily intake), seafood, low-fat cheese, chocolate (only dark chocolate, with more than 50% cocoa), and whole-grain cereals.

B. Control diet group. A low-fat, high complexcarbohydrate diet, as recommended by the National CholesterolEducation Program, with <30% of total calories from fat (12 -14%MUFAs, 6-8% PUFAs, < 10% SFAs), 55% from carbohydrates and 15% from protein. In both diets, the cholesterol content was adjusted to<300 mg/day (6). The focus in the control group was to reduce all types of fat, with

particular emphasis in recommending the consumption of lean meats, low-fat dairy products, cereals, potatoes, pasta, rice, fruits and vegetables.

Data collection/analysis

Evaluation of adherence to the Mediterranean and to the low-fat diet

For participants in the Mediterranean-diet group, researcher will undertake individual and group dietary-training sessions at the baseline visit and every three months thereafter. In each session, participants completed a 14-item dietary questionnaire to assess adherence to the Mediterranean diet so that personalized advice could be provided to the study participants in these groups.

Information relating to the dietary habits of the enrolled patients will be collected through a specific questionnaire (semiquantitative food frequency questionnaire (F.F.Q.s) (17) dedicated to the evaluation of the frequency of different food items in the diet and adapted to the Sicilian population. The subjects enrolled were classified based on the levels of adherence to a Mediterranean-style diet according to the methods proposed by Trichopoulou et al. (18).

The patients enrolled in the study will be assess for their adherence to a dietary regimen of the Mediterranean-style diet using the Mediterranean Diet Score, and the quantity and frequency of consumption of the food items characterising a Mediterranean-style diet were evaluated.

The consumption of foods presumed to be far from this diet pattern (i.e., rare or monthly consumption, meat and meat products, poultry and complete fat products) will be assign scores on an inverse scale. A value of 0 or 1 will be assign to each indicated food component. For the beneficial components, patients whose consumption was below the median were assigned a value of 0. For components presumed to be detrimental, patients whose consumption was below the median were assigned a value of 1.

We will apply a non-monotonous function for the alcohol, i.e., score 5 for the consumption of less than 300 ml of alcohol per day, score 0 for no consumption or for the consumption of > 700 ml per

day and scores from 4 to 1 for the consumption of 600-700, 500-600, 400-500 and 300-400 ml per day (100 ml is equivalent of 12 g ethanol).

Participants in the control group also will receive a dietary training at the baseline visit and completed the 10-item questionnaire at baseline to assess their adherence to the Mediterranean diet. During admission visit of the study, they received a leaflet explaining the low-fat diet on three month basis and assessing adherence to this low-fat diet with the use of a separate 9-item dietary questionnaire. Scores ranged from 0 to 9, with higher scores indicating greater adherence to a low-fat diet.

Biochemical Analysis

Blood samples will be obtained in the morning after at least ten fasting hours and will be stored by cryopreservation at -80°C.

Fasting plasma glucose levels, total cholesterol, triglycerides, HDL cholesterol, and LDL cholesterol were measured using traditional enzymatic methods at enrolment and at the 6- and 12-month follow-ups.

After 10 min of rest in the supine position, vital signs will be recorded, and blood samples were collected from the antecubital vein. EDTA-anticoagulated peripheral blood was drawn from each patient within 12 h of symptom onset. Serum and plasma were immediately separated by centrifugation and stored in aliquots at -80 °C until analysis.

Adiponectin, resistin and IL-6 serum levels will be measured by enzyme-linked immunosorbent assay (ELISA) according to the manufacturer's instructions. For adiponectin and resistin evaluation, high sensitivity kits (Biovendor) were used; IL-6 was determined by the Diaclone ELISA kit. Regarding the sensitivity of the adiponectin test (Biovendor), the analytical limit of detection was 0.6 microg/ml; the intra- and interassay coefficients of variation (%) were 4.1 and 4.0, respectively. For the resistin assay (Biovendor), the analytical limit of detection was 0.1 ng/ml. Visfatin was measured

by Sandwich ELISA (visfatin Phoenix Pharmaceuticals Inc.); the minimum detectable concentration for visfatin was 1.8 ng/ml.

Endothelial function evaluation

The pulse amplitude tonometry (PAT) probe will be placed on one finger of each of the two hands. After 5 minutes of control measurement, the pressure cuff was inflated to 200 mmHg for 5 minutes to induce reactive hyperaemia and then deflated.

The RH-PAT will be analysed at enrolment and at the 6- and 12-month follow-ups. Measurement was made digitally using the Endo-PAT2000 software version 3.0.4 device. The RH-PAT index reflects the extent of reactive hyperaemia. It will be calculated as the ratio of the average of the PAT signal amplitudes above the first minute of initial measurement to 1.5 minutes of measurement following deflation of the cuff (A: Control Arm; C: Occluded Arm) divided by the average of the PAT signal amplitudes over 2.5 minutes before inflation of the pressure cuff (B: Control Arm; D: Occluded Arm). This RH-PAT index, called the reactive hyperaemia index (RHI), is expressed by the formula RHI = (C/D)/(A/B) x basal correction.

Study samples and metabolomics profiling

All analyses will be performed by fasting (fasting for ≥8 hours) plasma EDTA samples collected at baseline and year 1. All samples will be processed at each recruiting centre no later than 2 hours after collection and stored in −80 °C freezers. Samples from cases and controla were randomly distributed before being shipped to the laboratory for metabolomics assays. LC-MS techniques were used to quantitatively profile ceramides in plasma samples. Plasma ceramide metabolites were measured concurrently with other lipid metabolites on the same platform and were identified based on total acyl carbon. Plasma ceramide concentrations (µg/mL) were assayed using a validated LC-MS/MS protocol at enrolment and at the 6- and 12-month follow-ups.

Plans for outcome assessment

The study hypothesis of our randomized parallel trial involves the evaluation of the hypothesized beneficial effect of adherence to a Mediterranean-style diet in subjects at high cardiovascular risk on surrogate vascular markers encompassing vascular health indices such as endothelial function indices and ceramide plasma pathways. Such markers could be regulated by amelioration of the lipidaemic profile and by modulation of markers of inflammatory adipose dysfunction such as adipokine serum levels.

Aims

- The first aim was to evaluate the effects of adherence to a Mediterranean-style diet on some vascular health indices, such as endothelial function and arterial stiffness markers.
- The second aim was to evaluate the effects of adherence to a Mediterranean-style diet on the lipidaemic profile and on some serum ceramide levels.
- The third aim was to analyse the effects induced by adherence to a Mediterranean-style dietary regimen on the modulation of serum concentrations of some adipokine serum levels.

References

1. Keys A et al. A Multivariate Analysis of Death and Coronary Heart Disease. Cambridge Mass, Harvard University Press, 1980; Pp. 1-381.
2. Definition of the Mediterranean Diet; a Literature Review. Nutrients. 2015;7(11)-9139-9153.
3. Buzina, R.; Keys, A.; Mohacek, I.; Marinkovic, M.; Hahn, A.; Blackburn, H. Coronary Heart Disease in Seven Countries. V. Five-Year Follow-up in Dalmatia and Slavonia.

4. de Lorgeril M, Salen P, Martin JL, Monjaud I, Delaye J, Mamelle N. Mediterranean diet, traditional risk factors, and the rate of cardiovascular complications after myocardial infarction: final report of the Lyon Diet Heart Study. Circulation. 1999 Feb 16;99(6):779-85.
5. Trichopoulou A, Costacou T, Bamia C, Trichopoulos D. Adherence to a Mediterranean diet and survival in a Greek population. N Engl J Med. 2003 Jun 26;348(26):2599-608
6. Ceriello A, Esposito K, La Sala L, Pujadas G, De Nigris V, Testa R, et al. The protective effect of the Mediterranean diet on endothelial resistance to GLP-1 in type 2 diabetes: a preliminary report. Cardiovasc Diabetol. 2014;13(1):140.
7. Estruch, R.; Ros, E.; et al. Primary Prevention of Cardiovascular Disease with a Mediterranean Diet Supplemented with Extra-Virgin Olive Oil or Nuts. N. Engl. J. Med. 2018, 378, E34.
8. Dimitriou, M.E.; Dedoussis, G.V.Z. Gene–Diet Interactions in Cardiovascular Di-Sease. Curr. Nutr. Rep. 2012, 1, 153–160.
9. Siri-Tarino PW, Sun Q, Hu FB, Krauss RM. Saturated fatty acids and risk of coronary heart disease: modulation by replacement nutrients. *Curr Atheroscler Rep*. 2010;12(6):384-390.
10. Siri-Tarino, Patty W et al. "Saturated fatty acids and risk of coronary heart disease: modulation by replacement nutrients." *Current atherosclerosis reports* vol. 12,6 (2010): 384-90.
11. Quintero-Flórez, A.; Sinausia Nieva, L.; et al. The Fatty Acid Composition of Virgin Olive Oil from Different Cultivars Is Determinant for Foam Cell Formation by Macrophages. J. Agric. Food Chem. 2015, 63, 6731–6738.
12. Chavez Jose A, Summers Scott A. A Ceramide-Centric View of Insulin Resistance. Cell Metab. 2012; 15:585–594.
13. Chavez Jose A, Summers Scott A. A Ceramide-Centric View of Insulin Resistance. Cell Metab. 2012; 15:585–594.
14. Summers SA. Ceramides in insulin resistance and lipotoxicity. Prog Lipid Res. 2006; 45:42–72; Summers Scott A. The ART of Lowering Ceramides. Cell Metab. 2015; 22:195–196.
15. Giroud M, Jodeleit H, Prentice KJ, Bartelt A. Adipocyte function and the development of cardiometabolic disease. J Physiol. 2021 Sep 23. doi: 10.1113/JP281979.
16. Laaksonen R, Ekroos K, Sysi-Aho M, Hilvo M, Vihervaara T, Kauhanen D, Suoniemi M, Hurme R, März W, Scharnagl H, Stojakovic T, Vlachopoulou E, Lokki ML, Nieminen MS, Klingenberg R, Matter CM, Hornemann T, Jüni P, Rodondi N, Räber L, Windecker S, Gencer B, Pedersen ER, Tell GS, Nygård O, Mach F, Sinisalo J, Lüscher TF. Plasma ceramides predict cardiovascular death in patients with stable coronary artery disease and acute coronary syndromes beyond LDL-cholesterol. Eur Heart J. 2016 Jul 1;37(25):1967-76
17. J.D. Fern_andez-Ballart, J.L. Pi~nol, I. Zazpe, et al., Relative Validity of a Semiquantitative Food-Frequency Questionnaire in an Elderly Mediterranean Population of Spain, Br. J. Nutr. 103 (2010) 1808e1816.
18. Trichopoulou A, Orfanos P, Norat T et al . Modified Medierranean Diet and Survival: EPIC-Elderly Prospective Cohort .Study. BMJ 2005 ; 330:99.
19. Tuttolomondo A, La Placa S, Di Raimondo D, Bellia C, Caruso A, Lo Sasso B, Guercio G, Diana G, Ciaccio M, Licata G, Pinto A. Adiponectin, resistin and IL-6 plasma levels in subjects with diabetic foot

and possible correlations with clinical variables and cardiovascular co-morbidity. Cardiovasc Diabetol. 2010 Sep 13;9:50.

20. Bonetti PO, Pumper GM, Higano ST, Holmes DR Jr, Kuvin JT, Lerman A. Noninvasive identification of patients with early coronary atherosclerosis by assessment of digital reactive hyperemia. J Am Coll Cardiol. 2004 Dec 7;44(11):2137-41
21. American Diabetes Association. Classification and Diagnosis of Diabetes: *Standards of Medical Care in Diabetes—2019* . Diabetes Care 2019 Jan; 42(Supplement 1): S13-S28
22. Williams B, Giuseppe Mancia, Wilko Spiering, Enrico Agabiti Rosei, Michel Azizi, Michel Burnier, Denis L Clement, Antonio Coca, Giovanni de Simone, Anna Dominiczak. 2018 ESC/ESH Guidelines for the management of arterial hypertension: The Task Force for the management of arterial hypertension of the European Society of Cardiology (ESC) and the European Society of Hypertension (ESH). *European Heart Journal*, Volume 39, Issue 33, 01 September 2018, Pages 3021–3104
23. Expert Panel on Detection, Evaluation, and Treatment of High Blood Cholesterol in Adults. Executive Summary of The Third Report of The National Cholesterol Education Program (NCEP) Expert Panel on Detection, Evaluation, And Treatment of High Blood Cholesterol In Adults (Adult Treatment Panel III). JAMA. 2001 May 16;285(19):2486-97.
24. Tzima N, Pitsavos C, Panagiotakos DB, Skoumas J, Zampelas A, Chrysohoou C, Stefanadis C. Mediterranean diet and insulin sensitivity, lipid profile and blood pressure levels, in overweight and obese people; the Attica study. Lipids Health Dis. 2007 Sep 19;6:22. doi: 10.1186/1476-511X-6-22.
25. Delgado-Lista J, Alcala-Diaz JF, Torres-Peña JD, Quintana-Navarro GM, Fuentes F, Garcia-Rios A, Ortiz-Morales AM, Gonzalez-Requero AI, Perez-Caballero AI, Yubero-Serrano EM, Rangel-Zuñiga OA, Camargo A, Rodriguez-Cantalejo F, Lopez-Segura F, Badimon L, Ordovas JM, Perez-Jimenez F, Perez-Martinez P, Lopez-Miranda J; CORDIOPREV Investigators. Long-term secondary prevention of cardiovascular disease with a Mediterranean diet and a low-fat diet (CORDIOPREV): a randomised controlled trial. Lancet. 2022 May 14;399(10338):1876-1885. doi: 10.1016/S0140-6736(22)00122-2.
26. Wang DD, Toledo E, Hruby A, Rosner BA, Willett WC, Sun Q, Razquin C, Zheng Y, Ruiz-Canela M, Guasch-Ferré M, Corella D, Gómez-Gracia E, Fiol M, Estruch R, Ros E, Lapetra J, Fito M, Aros F, Serra-Majem L, Lee CH, Clish CB, Liang L, Salas-Salvadó J, Martínez-González MA, Hu FB. Plasma Ceramides, Mediterranean Diet, and Incident Cardiovascular Disease in the PREDIMED Trial (Prevención con Dieta Mediterránea). Circulation. 2017 May 23;135(21):2028-2040.
