# Supplementary material for: Mediterranean diet effects on vascular health and serum levels of adipokines and ceramides
Source: PLoS One. 2024 May 29;19(5):e0300844. doi: 10.1371/journal.pone.0300844 (PMC11135776; doi:10.1371/journal.pone.0300844)
Supplement: Attachment — Submitted filename: response to reviewers.docx [file pone.0300844.s002.docx]

**Dear Dr. Tuttolomondo,**

**When submitting your revision, we need you to address these additional requirements.**

Dear Editor thank you for the evaluation of our manuscript and for the chance to submit a revised version of our manuscript

1. **Please ensure that your manuscript meets PLOS ONE's style requirements, including those for file naming. The PLOS ONE style templates can be found at
   https://journals.plos.org/plosone/s/file?id=wjVg/PLOSOne_formatting_sample_main_body.pdf and** 
   [**https://journals.plos.org/plosone/s/file?id=ba62/PLOSOne_formatting_sample_title_authors_affiliations.pdf**](https://journals.plos.org/plosone/s/file?id=ba62/PLOSOne_formatting_sample_title_authors_affiliations.pdf)

We ensured it

**2. When completing the data availability statement of the submission form, you indicated that you will make your data available on acceptance. We strongly recommend all authors decide on a data sharing plan before acceptance, as the process can be lengthy and hold up publication timelines. Please note that, though access restrictions are acceptable now, your entire data will need to be made** freely accessible if your manuscript is accepted for publication. This policy applies to all data except where public deposition would **breach compliance with the protocol approved by your research ethics board. If you are unable to adhere to our open data policy, please kindly revise your statement to explain your reasoning and we will seek the editor's input on an exemption. Please be assured that, once you have provided your new statement, the assessment of your exemption will not hold up the peer review process.**

We upadated and corrected our data sharing

**3. PLOS requires an ORCID iD for the corresponding author in Editorial Manager on papers submitted after December 6th, 2016. Please ensure that you have an ORCID iD and that it is validated in Editorial Manager. To do this, go to ‘Update my Information’ (in the upper left-hand corner of the main menu), and click on the Fetch/Validate link next to the ORCID field. This will take you to the ORCID site and allow you to create a new iD or authenticate a pre-existing iD in Editorial Manager. Please see the following video for instructions on linking an ORCID iD to your Editorial Manager account: https://www.youtube.com/watch?v=_xcclfuvtxQ**

**4. Please include a separate caption for each figure in your manuscript.**

We included a caption for each figure in our manuscript

**5. Please include a copy of Table 2 which you refer to in your text on page 16.**

**Table 2 is present on the text now**

**6. Please include captions for your Supporting Information files at the end of your manuscript, and update any in-text citations to match accordingly. Please see our Supporting Information guidelines for more information:** [**http://journals.plos.org/plosone/s/supporting-information**](http://journals.plos.org/plosone/s/supporting-information)**.**

**We included the captions**

**Reviewers' comments:

Dear Editor,**

**Thank you very much for the useful revision comments and for the chance to submit a revised version of our manuscript.**

**You will find above our answers to the reviewer comments.**

5. Review Comments to the Author

Please use the space provided to explain your answers to the questions above. You may also include additional comments for the author, including concerns about dual publication, research ethics, or publication ethics. (Please upload your review as an attachment if it exceeds 20,000 characters)

**Reviewer #1: The manuscript addresses an interesting topic. The collected data are unique, the employed statistical methods rather basic and there is room left for improvements. My main comments are indeed on the performed statistical analysis.**

**1. It is nice to see that data are attached. Please, provide the code use to obtain the results in the tables as well.
Nevertheless, I feel unacceptable that the data are not anonymized. All patients information, also phone numbers, are available to anyone with access to the link in the paper. This is a clear violation of the privacy and it is rather sloppy and annoying.**

As indicated by the reviewer we removed the information available in the previous version of the attached data that have been enclosed only for a mistake.

**2. The use of parametric tests is in general sound, but all tests are based on quite strong assumptions. In the main text, the authors state that they check for these assumptions, please provide evidence that they are met.**

Dear reviewer thanks for your interesting comments that could be important for the verification of the assumption of the observed significance. We have performed for all variables, in particular with regard of the quantitative variables, the test of normality of the data by kolmogorov-Smirnov test with Lilliefors correction. We found non-significant comforting results about the normality of the data and not relying only on the kolmogorov-Smirnov test. Thus, we repeated the data analysis also with nonparametric statistical procedures that provided identical results with the same significance emerged. Moreover, further sustainment concerning the results that have emerged can also be attributed to the fact that we compared patients at different study times through the paired data methodology, and this seemed to us to be further evidence of the goodness of fit of the data. Therefore, we have chosen to present the data only as means by reporting the parametric results according to ANOVA analysis.This choice is linked to our willness to not furtherly burden the text by reporting all the normality significances for the different variables.

3**. The employed statistical methods are too basic. I strongly suggest to consider random effects regression models to better account for the longitudinal structure of the data. As before, a residual analysis should be performed and discussed to ensure the reliability of the results. Instead of looking at one variable at the time, a full model should be considered and variable selection procedure implemented and discussed. I further suggest to discuss the potential impact of missing values, which are likely to arise in studies of this type (and it is not clear if any missing values is present in the current study too).**

We thank the reviewer again for this additional comment. Indeed in a previous draft of the manuscript we have had wondered of including some regression analyses that , also on the light of your suggestions, we have now decided to resubmit. It seemed to us that the results obtained and presented in the two tables were already sufficient to demonstrate the objectives of the study, but indeed the multivariable regression model for the variables seem to result adequate to be furtherly analyzed since lipid profile, markers of vascular damage such as RHI and AIX, and serum levels of visfatin, adiponectin, and resistin, could furtherly emphasizes the role of these clinical variables as markers of efficacy of the Mediterranean diet in the studied subjects. On the other hand, regarding the request for missing data, since this is a one-year study (6- and 12-month follow-ups) and not a large number of participants, we did not have a drop out of patients and the completeness of the collected data exceeds 98% for all variables. Consequently, we do not believe that this issue could have affected the results of our study.

**Reviewer #2: The study titled "Mediterranean diet effects on vascular health and serum levels of adipokines and ceramides" conducted by Daidone and colleagues evaluated the effect of Mediterranean diet on serum levels of adipokines and ceramides. The study is not well-designed. The topic is not novel and the way of presentation of results are not accurate. The English language needs extensive revisions by a native**

Dear reviewer

Thank you for your comments

In our answers to reviewer 1 we added some further information on the statistical analysis and on the design of the study

With regard of the issue of novelty as expressed on the text the findings of this prospective study concerning the observed positive effects of adherence to a Mediterranean-style diet on adipokine serum levels, ceramide serum levels and an index of vascular health, such as RHI, may help to explain the cardiovascular and cerebrovascular protective relationship reported in previous studies from our own group with regard to ischaemic stroke, in particular, the atherosclerotic subtype (65), hamorrhagic cerebrovascular disorders (66) and congestive heart failure (67-69). This integrated evaluation is new, whereas the effects of a Mediterranean Diet on ceramide and adipokine serum levels it is novel finding.

We furtherly revised the English style of the manuscript by a mother tongue revisor (see the Editing certification)

**Reviewer #3: Daidone et al. have performed a study on the association of the Mediterranean diet with vascular health and serum adipokines. The study is well-conducted and well-written. I have some comments to consider:

- The introduction section is unnecessarily lengthy. The authors should try to focus on the main ideas and the rationale for performing the study.**

Dear reviewer thanks for your interesting comment. As suggested, we completely revised the introduction with the aim of making the paragraph more focused on the background that led us to design this study.

**- Figure 1 is not completed as the numbers are not added to the flowchart.**

Dear reviewer, “see figure 1” has been written by mistake. We provided to delete it from the definitive manuscript

**- The first paragraph of the discussion should focus on the main findings of the manuscript. It should be revised.**

Thanks again for your suggestion. We added a little paragraph in which are synthetizes the main findings of our study.

6. PLOS authors have the option to publish the peer review history of their article ([what does this mean?](https://journals.plos.org/plosone/s/editorial-and-peer-review-process#loc-peer-review-history)). If published, this will include your full peer review and any attached files.


If you choose “no”, your identity will remain anonymous but your review may still be made public.


**Do you want your identity to be public for this peer review?** For information about this choice, including consent withdrawal, please see our [Privacy Policy](https://www.plos.org/privacy-policy).

Reviewer #1: No

Reviewer #2: No

Reviewer #3: No
